# Ultrasonographic measurements of fascicle length overestimate adaptations in serial sarcomere number

**DOI:** 10.1113/EP091334

**Published:** 2023-08-23

**Authors:** Avery Hinks, Martino V. Franchi, Geoffrey A. Power

**Affiliations:** ^1^ Department of Human Health and Nutritional Sciences, College of Biological Sciences University of Guelph Guelph Ontario Canada; ^2^ Department of Biomedical Sciences, Human Neuromuscular Physiology Laboratory University of Padua Padua Italy; ^3^ CIR‐MYO Myology Centre University of Padua Padua Italy

**Keywords:** casting, fascicle, immobilization, pennation angle, sarcomere, sarcomerogenesis, ultrasound

## Abstract

Ultrasound‐derived measurements of muscle fascicle length (FL) are often used to infer increases (chronic stretch or training) or decreases (muscle disuse or aging) in serial sarcomere number (SSN). Whether FL adaptations measured via ultrasound can truly approximate SSN adaptations has not been investigated. We casted the right hindlimb of 15 male Sprague–Dawley rats in a dorsiflexed position (i.e., stretched the plantar flexors) for 2 weeks, with the left hindlimb serving as a control. Ultrasound images of the soleus, lateral gastrocnemius (LG), and medial gastrocnemius (MG) were obtained with the ankle at 90° and full dorsiflexion for both hindlimbs pre and post‐cast. Following post‐cast ultrasound measurements, legs were fixed in formalin with the ankle at 90°, then muscles were dissected and fascicles were teased out for measurement of sarcomere lengths via laser diffraction and calculation of SSN. Ultrasound detected an 11% increase in soleus FL, a 12% decrease in LG FL, and an 8–11% increase in MG FL for proximal fascicles and at full dorsiflexion. These adaptations were partly reflected by SSN adaptations, with a 6% greater soleus SSN in the casted leg than the un‐casted leg, but no SSN differences for the gastrocnemii. Weak relationships were observed between ultrasonographic measurements of FL and measurements of FL and SSN from dissected fascicles. Our results showed that ultrasound‐derived FL measurements can overestimate an increase in SSN by ∼5%. Future studies should be cautious when concluding a large magnitude of sarcomerogenesis from ultrasound‐derived FL measurements, and may consider applying a correction factor.

## Abstract (highlights)

- **What is the central question of this study?**
  Measurements of muscle fascicle length via ultrasound are often used to infer changes in serial sarcomere number, such as increases following chronic stretch or resistance training, and decreases with ageing: does ultrasound‐derived fascicle length accurately depict adaptations in serial sarcomere number?
- **What is the main finding and its importance?**
  Ultrasound detected an ∼11% increase in soleus fascicle length, but measurements on dissected fascicles showed the actual serial sarcomere number increase was only ∼6%; therefore, measurements of ultrasound‐derived fascicle length can overestimate serial sarcomere number adaptations by as much as 5%.

## 1 INTRODUCTION

Characterization of a muscle's serial sarcomere number (SSN) gives insight into properties of biomechanical function (Hinks, Franchi et al., 2022; Lieber & Fridén, 2000; Narici et al., 2016). To that end, B‐mode ultrasound is often used in humans to measure fascicle length (FL) and infer SSN adaptations at a smaller scale, such as increases in FL following resistance training (Blazevich et al., 2007; Franchi et al., 2014; Hinks et al., 2021) or decreases in FL with age and disuse (de Boer et al., 2008; Narici et al., 2003; Power et al., 2013; Williams & Goldspink, 1978). In animals, SSN can be estimated more precisely by dividing the length of a dissected fascicle by the average sarcomere length (SL) measured via laser diffraction (Butterfield et al., 2005; Chen et al., 2020; Hinks, Jacob et al., 2022). Unfortunately, direct measurement of SL in humans is invasive (Boakes et al., 2007; Lieber et al., 1997), and often prohibitively costly and not accessible (Adkins et al., 2021; Lichtwark et al., 2018). However, inferring SSN adaptations via ultrasound‐derived measurements of FL may be problematic because apparent increases or decreases in FL could be due to longer or shorter SLs, respectively, at the joint angle in which FL was measured (Pincheira et al., 2021). The relationship between SSN and FL may also depend on the region of muscle, an example being the human tibialis anterior displaying greater SSN in proximal fascicles due to a shorter SL (Lichtwark et al., 2018). Collectively, the relationship between SSN and ultrasound‐derived FL may depend on the joint angle and region of muscle at which measurements are taken. Whether FL adaptations measured via ultrasound truly approximate SSN adaptations has not been investigated.

Assessment of FL in rodents via ultrasound is less common than in humans, but not unfounded. Peixinho and colleagues developed reliable methods for assessment of muscle architecture via ultrasound in the rat plantar flexors (Peixinho et al., 2011, 2014). Ultrasonography of the rat plantar flexors also has enough sensitivity to detect morphological adaptations (Mele et al., 2016; Peixinho et al., 2014). These previous studies, however, only assessed pennation angle (PA) and muscle thickness, leaving characterization of ultrasound‐derived FL adaptations in rats unclear. Altogether, rodent models present an opportunity to assess the sensitivity of ultrasound measurements of FL in detecting actual SSN adaptations.

The present study assessed the validity of ultrasound as a tool to detect adaptations in SSN. To do this, we immobilized the rat plantar flexors in a lengthened position – an intervention that rapidly increases soleus SSN (Aoki et al., 2009; Soares et al., 2007; Tabary et al., 1972; Williams & Goldspink, 1978). We hypothesized that the ability for ultrasound‐derived FL measurements to characterize adaptations in SSN would vary depending on the joint angle at which ultrasound measurements are obtained and the region of muscle.

## 2 METHODS

### 2.1 Ethical approval

Approval was given by the University of Guelph's Animal Care Committee (AUP no. 4905) and all protocols followed the Canadian Council on Animal Care guidelines.

### 2.2 Animals

Fifteen male Sprague–Dawley rats (age at killing ∼19 weeks) were obtained (Charles River Laboratories, Senneville, QC, Canada). Rats were housed at 23°C in groups of three and given ad libitum access to a Teklad global 18% protein rodent diet (Envigo, Huntington, UK) and room‐temperature water. The right ankle was immobilized in near‐maximal dorsiflexion for 2 weeks to place the plantar flexor muscles, in particular the soleus, in a lengthened position (Aoki et al., 2009; Soares et al., 2007). Per previous investigations of SSN adaptations in immobilized rat muscle, the contralateral limb served as a control (Gomes et al., 2004; Heslinga & Huijing, 1993). Ultrasound images of the lateral gastrocnemius (LG), medial gastrocnemius (MG) and soleus were obtained at ∼17 weeks of age (pre‐immobilization) and ∼19 weeks of age (post‐immobilization). All anaesthetic procedures (ultrasound image acquisition and application of casts) were conducted via isoflurane inhalation, using a flow rate of 1.5 litres/min with 2.5% isoflurane.

### 2.3 Unilateral immobilization

**FIGURE 1:**
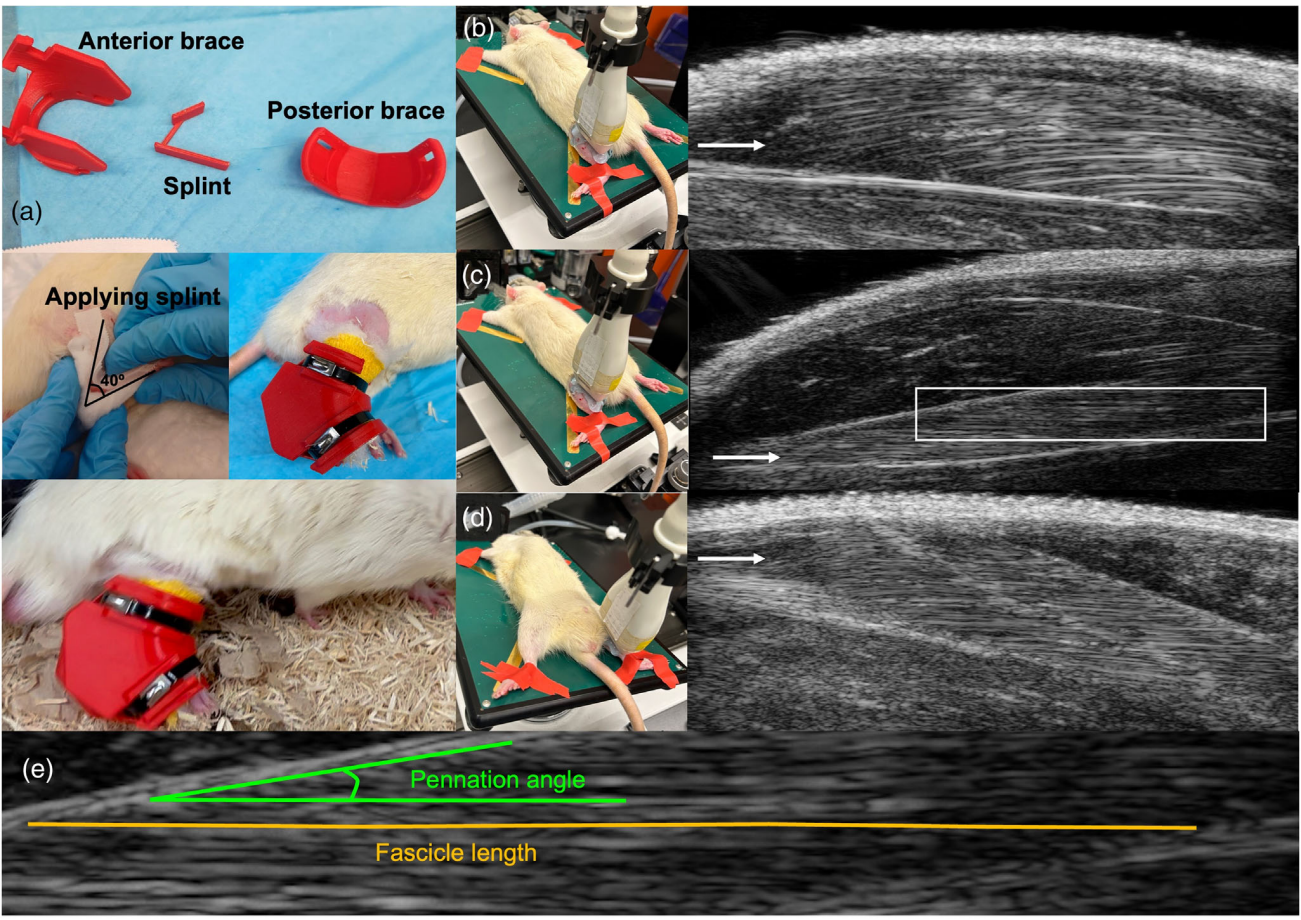
(a) Example images of applying the splint and brace for the dorsiflexion cast. (b–d) Set‐up and example of ultrasound images obtained from the left lateral gastrocnemius (b), soleus (c) and medial gastrocnemius (d), with the ankle fixed at 90° using tape. White arrows indicate the muscle of interest in each image. (e) The area highlighted by the white box in (c), showing representative tracings of fascicle length (orange) and pennation angle (green).

Using gauze padding, vet wrap, and a 3D‐printed brace and splint, the right hindlimb of each rat was immobilized in dorsiflexion (40° ankle angle; full plantar flexion = 180°) (Figure 1a). Casts were inspected daily and repaired/replaced as needed. The toes were left exposed to monitor for swelling (Aoki et al., 2009).

### 2.4 Ultrasonography

Ultrasound measurements were obtained from the right and left hindlimbs at pre‐immobilization (no more than 1 week prior to first applying the casts) and post‐immobilization (immediately following cast removal).

A UBM system (Vevo 2100; VisualSonics, Toronto, ON, Canada) operating at a centre frequency of 21 MHz was used to acquire images of the soleus, LG and MG, with a lateral resolution of 80 μm and an axial resolution of 40 μm (Mele et al., 2016). A 23‐mm‐long probe was used, allowing acquisition of images displaying muscle fascicles from end to end. During piloting, image acquisition was optimized with an image depth of 15 mm for the soleus and LG and 16 mm for the MG, both allowing a maximum frame rate of 16 Hz. Prior to image acquisition, rats were anaesthetized using isoflurane. With the knee fully extended, tape was used to fix the ankles at two different positions for image acquisition: (1) 90° and (2) full dorsiflexion. All ultrasound images were acquired by the same individual (A.H.). Images of the LG and soleus were obtained with the rat in a prone position and the hindlimb externally rotated, with the probe overlying the lateral aspect of the posterior shank (Figure 1b,c). Images of the MG were obtained with the rat in a supine position and the hindlimb externally rotated, with the probe overlying the medial aspect of the posterior shank (Figure 1d). The probe position was carefully adjusted to obtain the clearest possible view of fascicles in all of the proximal, middle and distal regions of the muscle. Throughout image acquisition, the probe was stabilized by a crane with fine‐tune adjustment knobs, minimizing pressure and limiting the error associated with human movement.

Ultrasound images were analysed using ImageJ software (Franchi et al., 2019). ImageJ's multisegmented tool allowed careful tracing of the fascicle paths from end to end in measuring FL. Two measurements of FL and PA were obtained from each of the proximal, middle and distal regions of each muscle (i.e., *n* = 6 FL and PA measurements per muscle) and averaged for each region for the reporting of data (i.e., *n* = 1 averaged measurement at each of the proximal, middle and distal regions). PA was defined as the angle between the fascicle and the aponeurosis at the fascicle's distal insertion point. All FL and PA measurements were obtained by the same experimenter (A.H.), who was blinded to the results until all measurements pre‐ and post‐immobilization were obtained. During piloting, across three separate image acquisitions on the same rat, the coefficients of variation (standard deviation/mean × 100%) for FL averaged among two measurements at each region of muscle were all <10% (Table 1), which indicates low variation among repeated measures.

**TABLE 1:** Coefficients of variation for fascicle length across three separate image acquisitions on the same rat (*n* = 1).

Day	Proximal FL 1 (mm)	Proximal FL 2 (mm)	Proximal FL average (mm)	Middle FL 1 (mm)	Middle FL 2 (mm)	Middle FL average (mm)	Distal FL 1 (mm)	Distal FL 2 (mm)	Distal FL average (mm)	Total FL average (mm)
Lateral gastrocnemius										
1	11.79	11.79	**11.79**	12.96	13.10	**13.03**	12.69	12.00	**12.34**	**12.39**
2	10.25	12.75	**11.50**	12.78	13.59	**13.19**	12.26	13.68	**12.97**	**12.55**
3	12.56	11.30	**11.93**	12.61	13.03	**12.82**	14.56	12.85	**13.70**	**12.82**
CV (%)			**1.88**			**1.43**			**5.23**	**1.72**
Soleus										
1	10.27	10.69	**10.48**	11.82	12.11	**11.97**	10.62	10.12	**10.37**	**10.94**
2	10.66	10.66	**10.66**	12.51	10.58	**11.55**	11.12	9.73	**10.42**	**10.88**
3	10.60	10.44	**10.52**	11.29	10.43	**10.86**	10.79	10.39	**10.59**	**10.66**
CV (%)			**0.91**			**4.88**			**1.10**	**1.37**
Medial gastrocnemius										
1	10.52	11.93	**11.23**	12.42	13.47	**12.94**	13.06	12.50	**12.50**	**12.32**
2	11.11	12.36	**11.74**	12.33	13.56	**12.95**	13.45	12.91	**13.18**	**12.62**
3	10.66	10.88	**10.77**	11.86	13.60	**12.73**	13.76	12.31	**13.04**	**12.18**
CV (%)			**4.31**			**0.99**			**2.80**	**1.84**

Abbreviation: FL, fascicle length.

### 2.5 Serial sarcomere number estimations

Following the post‐immobilization ultrasound measurements, rats were killed via isoflurane anaesthetization followed by CO_2_ asphyxiation. The hindlimbs were amputated and fixed in 10% phosphate‐buffered formalin with the ankle pinned at 90° and the knee fully extended. After fixation for 1–2 weeks, the muscles were dissected and rinsed with phosphate‐buffered saline. The muscles were then digested in 30% nitric acid for 6–8 h to remove connective tissue and allow for individual muscle fascicles to be teased out (Butterfield et al., 2005; Hinks, Jacob et al., 2022).

For each muscle, two fascicles were obtained from each of the proximal, middle and distal regions of the muscle (i.e., *n* = 6 fascicles total per muscle) and averaged for each region for the reporting of data (i.e., *n* = 1 averaged measurement at each of the proximal, middle and distal regions). Dissected fascicles were placed on glass microslides (VWR International, Mississauga, Ontario, Canada), then FLs were measured using ImageJ software (version 1.53f, NIH, Bethesda, MD, USA) from pictures captured by a level, tripod‐mounted digital camera, with measurements calibrated to a ruler in plane with the fascicles (Supporting information, Figure S1). Sarcomere length measurements were taken at *n* = 6 different locations proximal to distal along each fascicle via laser diffraction (Coherent, Santa Clara, CA, USA) with a 5‐mW diode laser (25 μm beam diameter, 635 nm wavelength) and custom LabVIEW program (Version 2011, National Instruments, Austin, TX, USA) (Lieber et al., 1984), for a total of *n* = 36 sarcomere length measurements per muscle. For each fascicle, the six SL measurements were averaged to obtain a value of average SL. Serial sarcomere number was calculated as: serial sarcomere number = fascicle length/mean sarcomere length.

### 2.6 Statistical analysis

Statistical analyses were conducted using GraphPad Prism 9.5.1 (GraphPad Software, San Diego, CA, USA). To investigate variation in ultrasound‐derived FL and PA, three‐way analysis of variance (ANOVA) (time [pre‐immobilization, post‐immobilization] × joint position [90°, full dorsiflexion] × region [proximal, middle, distal]) was performed for each muscle from each leg, with the Greenhouse–Geisser correction for sphericity. For each dissected muscle, a two‐way ANOVA (leg [casted, un‐casted] × region [proximal, middle distal]) was used to investigate variation in SSN, SL and FL, with the Greenhouse–Geisser correction for sphericity. For all ANOVAs, where interactions or effects of region were detected, pairwise comparisons (Student's two‐tailed paired *t*‐test) were performed with a Bonferonni correction for multiplicity. The two‐tailed, paired *t*‐test was used to compare muscle wet weights between the casted and un‐casted leg, with a Bonferroni correction for multiplicity. For all significant *t*‐tests, the effect size was reported as Cohen's *d*. Significance was set at α = 0.05.

Linear regression was used to investigate the relationship between: (1) ultrasound‐derived FL at 90° post‐cast and FL of dissected fascicles; (2) ultrasound‐derived FL at each joint angle post‐cast and SSN of dissected fascicles; and (3) adaptations in ultrasound‐derived FL (as % change pre to post‐cast) at each joint angle and adaptations in SSN of dissected fascicles (as % change from the un‐casted to the casted leg).

## 3 RESULTS

### 3.1 Effects of region, joint position and time on fascicle length measured via ultrasound

Three‐way ANOVA results for FL measured via ultrasound are presented in Table 2.

**TABLE 2:** Three‐way ANOVA results for ultrasound‐derived fascicle length (*n* = 15 rats).

		Effect of region		Effect of joint position		Effect of time		Region × joint position interaction		Region × time interaction		Joint position × time interaction		Region × joint position × time interaction	
		*F*	*P*	*F*	*P*	*F*	*P*	*F*	*P*	*F*	*P*	*F*	*P*	*F*	*P*
LG	Un‐casted	393.80	<0.0001*	215.10	<0.0001*	3.99	0.0819	3.01	0.0655	0.08	0.921	17.79	0.0014*	0.01	0.970
	Casted	271.00	<0.0001*	269.10	<0.0001*	50.10	<0.0001*	2.43	0.106	3.02	0.0649	0.60	0.414	3.08	0.0954
Soleus	Un‐casted	10.49	0.0022*	148.90	<0.0001*	0.09	0.677	3.39	0.0480*	2.07	0.145	1.13	0.296	0.67	0.426
	Casted	24.83	<0.0001*	236.80	<0.0001*	27.64	0.0003*	0.15	0.861	10.57	0.0004*	0.17	0.662	0.26	0.629
MG	Un‐casted	101.20	<0.0001*	99.57	<0.0001*	1.43	0.240	1.39	0.266	8.75	0.0011*	2.70	0.132	1.69	0.214
	Casted	120.30	<0.0001*	159.80	<0.0001*	3.62	0.0844	1.03	0.369	6.91	0.0036*	14.30	0.0046*	4.13	0.0596

Abbreviations: LG, lateral gastrocnemius; MG, medial gastrocnemius. *Significant effect or interaction (*P* < 0.05).

**FIGURE 2:**
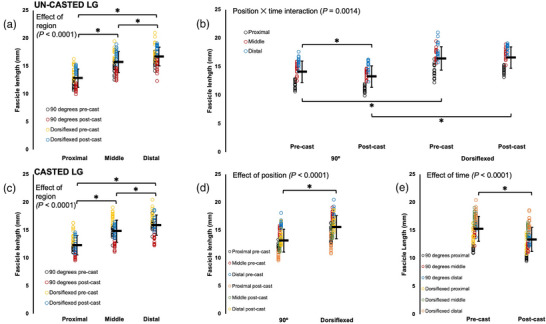
Fascicle length of the un‐casted and casted lateral gastrocnemius (LG) measured via ultrasound from *n* = 15 rats. For the un‐casted LG, there was an effect of region (a) and an interaction between joint position and time (b). For the casted LG, there were effects of region (c), joint position (d) and time (e). Data are presented as means ± standard deviation. *Significant difference between indicated means (*P* < 0.05).

**FIGURE 3:**
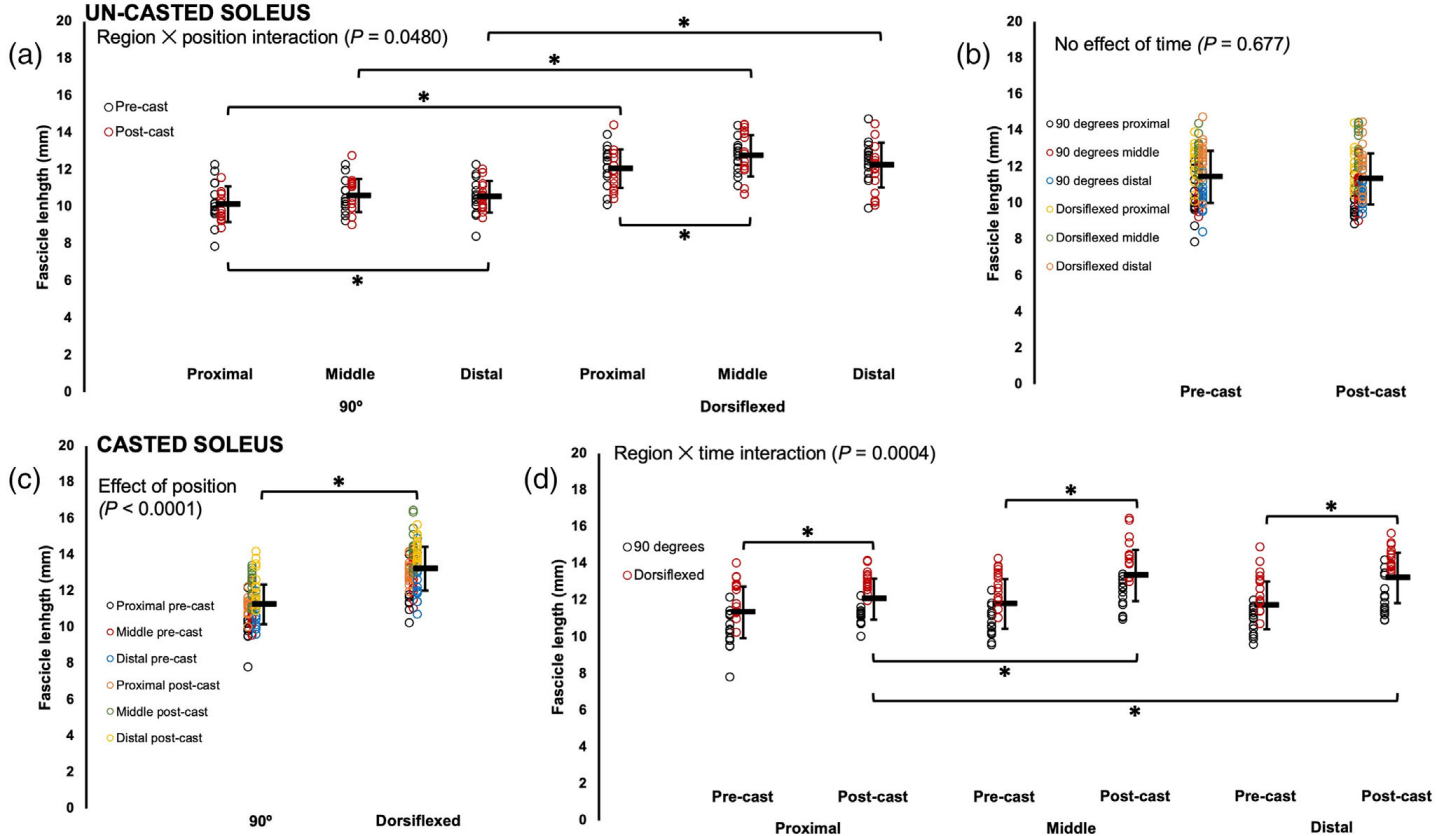
Fascicle length of the un‐casted and casted soleus measured via ultrasound from *n* = 15 rats. For the un‐casted soleus, there was an interaction between region and position (a) and no effect of time (b). For the casted soleus, there was an effect of position (c) and an interaction between region and time (d). Data are presented as means ± standard deviation. *Significant difference between indicated means (*P* < 0.05).

**FIGURE 4:**
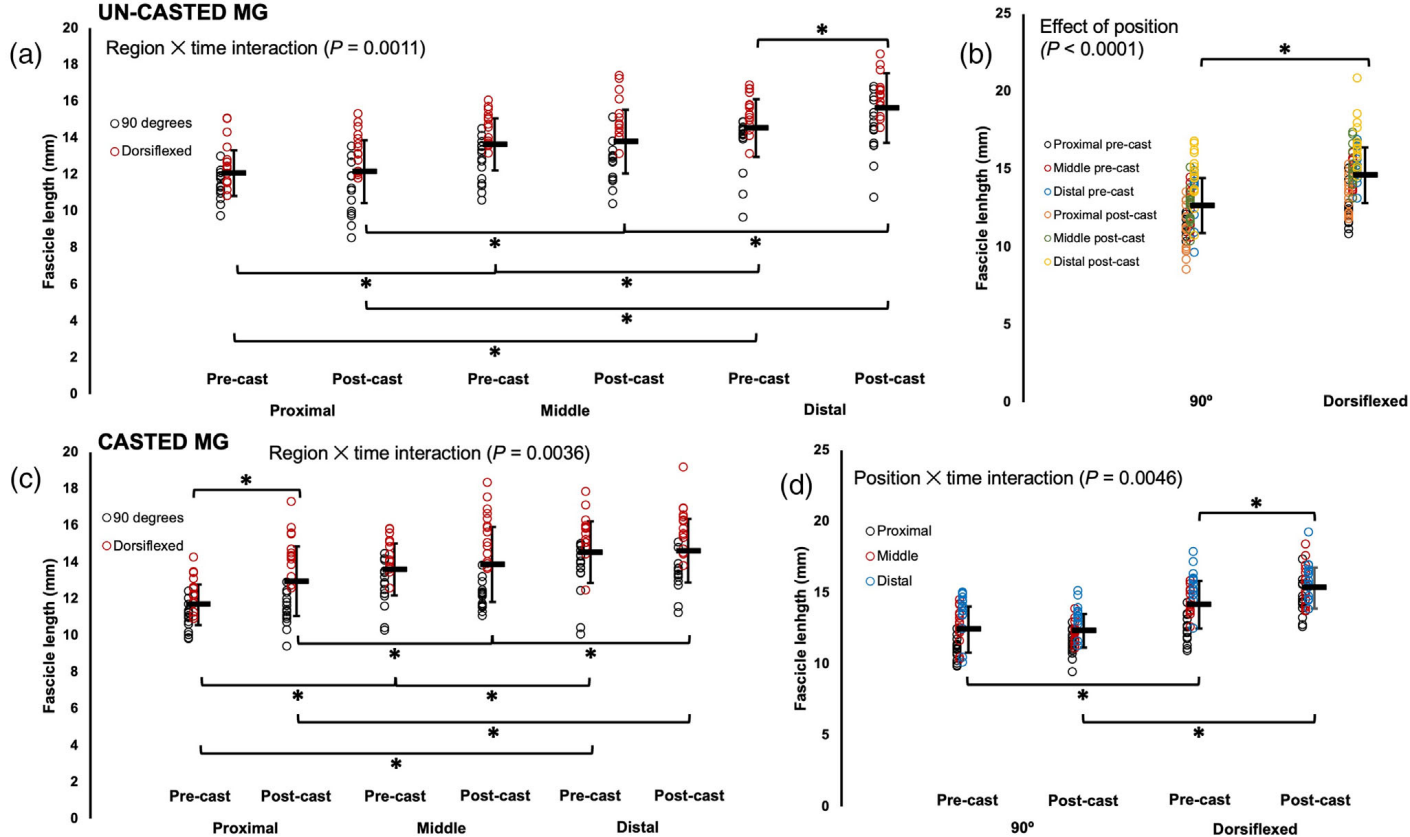
Fascicle length of the un‐casted and casted medial gastrocnemius (MG) measured via ultrasound from *n* = 15 rats. For the un‐casted MG, there was an interaction between region and time (a) and an effect of position (b). For the casted MG, there were interactions between region and time (c) and position and time (d). Data are presented as means ± standard deviation. *Significant difference between indicated means (*P* < 0.05).

For all muscles, there were effects of joint position, with FL increasing from a 90° ankle angle to full dorsiflexion (Table 2; Figures 2, 3, 4). For the gastrocnemii, there were effects of region, with FL increasing from proximal to distal (Table 2; Figures 2 and 4).

For ultrasound‐derived FL of the un‐casted LG, there was a joint position × time interaction (Table 2). Pairwise comparisons showed that FL decreased by 6% pre to post‐cast when measurements were performed at 90° (*P* = 0.0001, *d* = 0.45), but did not change according to measurements performed at full dorsiflexion (*P* = 1.00) (Figure 2b).

For ultrasound‐derived FL of the casted LG, there was an effect of time (Table 2), with FL decreasing by 12% pre to post‐cast (Figure 2c).

For ultrasound‐derived FL of the un‐casted soleus, there was a region × joint position interaction (Table 2). Pairwise comparisons showed distal fascicles were longer than proximal fascicles when measured at 90° (*P* = 0.0413, *d* = 0.44), but proximal and middle FL did not differ (*P* = 0.194), and middle and distal FL did not differ (*P* = 1.00) (Figure 3a). Conversely, in measurements performed at full dorsiflexion, middle fascicles were longer than proximal fascicles (*P* = 0.0003, *d* = 0.65), but proximal and distal FL did not differ (*P* = 1.00), and middle and distal FL did not differ (*P* = 0.0591) (Figure 3a). FL of the un‐casted soleus did not change from pre to post‐cast, with no effect of time (Figure 3b).

For the casted soleus, an effect of time showed that ultrasound‐derived FL increased on average by 11% pre to post‐cast (Table 2). There was also a region × time interaction. Pairwise comparisons showed all regions of the soleus increased FL from pre to post‐cast (proximal: *P* = 0.0301, *d* = 0.57; middle: *P* < 0.0001, *d* = 1.12; distal: *P* < 0.0001, *d* = 1.13) (Figure 3d). Pre‐cast, there were no regional differences in FL (*P* = 0.0849–1.00), but post‐cast, middle (*P* < 0.0001; *d* = 1.01) and distal fascicles (*P* < 0.0001; *d* = 0.91) were longer than proximal fascicles (Figure 3d). Accordingly, the increase in proximal FL from pre to post‐cast was smaller (+6%) than the increases in middle and distal FL (both +13%).

For ultrasound‐derived FL of the un‐casted MG, there was a region × time interaction (Table 2), with distal FL increasing by 8% pre to post‐cast (*P* = 0.0330, *d* = 0.63) (Figure 4a).

For ultrasound‐derived FL of the casted MG, there was also a region × time interaction (Table 2), but with proximal FL increasing by 11% pre to post‐cast (*P* = 0.0028, *d* = 0.82) (Figure 4c). A joint position × time interaction showed that measurements at 90° detected no change in FL pre to post‐cast (*P* = 1.00), but measurements at full dorsiflexion detected an 8% increase in FL (*P* = 0.0002, *d* = 0.76) (Figure 4d).

In summary, the casted soleus experienced an increase in ultrasound‐derived FL while the un‐casted soleus experienced no change. The casted LG experienced a decrease in ultrasound‐derived FL. A decrease in ultrasound‐derived FL was also observed in the un‐casted LG, but only in measurements at 90°. For the casted MG, there were increases in ultrasound‐derived FL in proximal fascicles and at full dorsiflexion. There was also an increase in ultrasound‐derived FL in the un‐casted MG in distal fascicles.

### 3.2 Effect of time on pennation angle measured via ultrasound

Three‐way ANOVA results for PA measured via ultrasound are presented in Table 3.

**TABLE 3:** Three‐way ANOVA results for ultrasound‐derived pennation angle (*n* = 15 rats).

		Effect of region		Effect of joint position		Effect of time		Region × joint position interaction		Region × time interaction		Joint position × time interaction		Region × joint position × time interaction	
		*F*	*P*	*F*	*P*	*F*	*P*	*F*	*P*	*F*	*P*	*F*	*P*	*F*	*P*
LG	Un‐casted	371.09	<0.0001*	101.59	<0.0001*	69.40	<0.0001*	44.15	<0.0001*	2.32	0.117	2.69	0.126	0.43	0.568
	Casted	274.65	<0.0001*	147.66	<0.0001*	55.73	<0.0001*	35.91	<0.0001*	32.54	<0.0001*	0.63	0.391	12.81	0.0013*
Soleus	Un‐casted	245.15	<0.0001*	163.54	<0.0001*	0.24	0.570	4.02	0.0292*	1.18	0.321	1.16	0.285	0.46	0.543
	Casted	73.30	<0.0001*	85.29	<0.0001*	122.94	<0.0001*	1.05	0.362	20.55	<0.0001*	20.29	0.0009*	2.28	0.144
MG	Un‐casted	132.70	<0.0001*	140.88	<0.0001*	0.81	0.357	4.51	0.0201*	3.13	0.0593	1.37	0.260	0.11	0.767
	Casted	139.24	<0.0001*	182.18	<0.0001*	80.12	<0.0001*	2.02	0.152	3.34	0.0499*	0.20	0.634	1.28	0.278

Abbreviations: LG, lateral gastrocnemius; MG, medial gastrocnemius. *Significant effect or interaction (*P* < 0.05).

**FIGURE 5:**
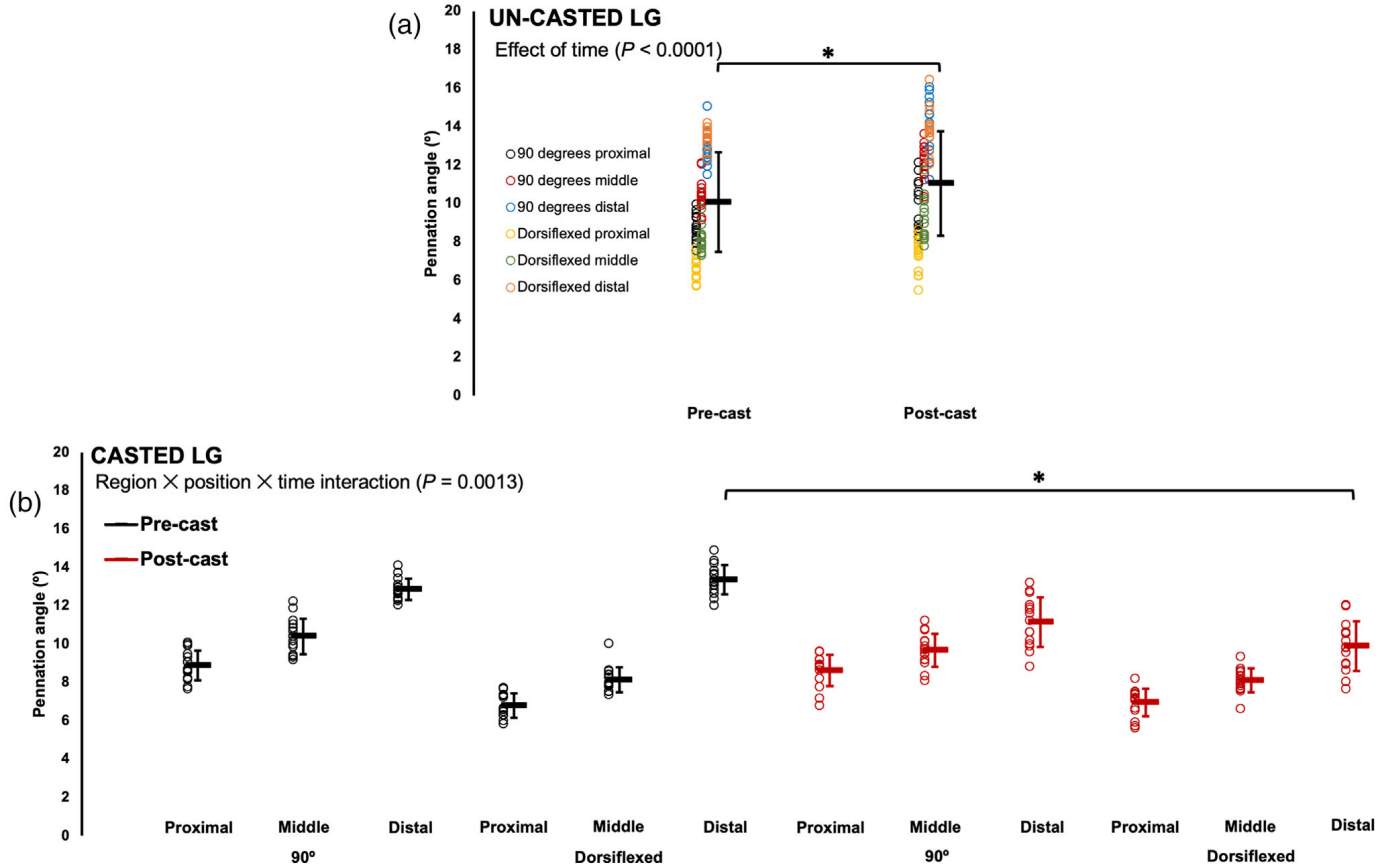
Changes in pennation angle of the un‐casted (a) and casted (b) lateral gastrocnemius (LG) from pre to post‐cast in *n* = 15 rats. Data are presented as means ± standard deviation. *Significant difference between indicated means (*P* < 0.05).

For the un‐casted LG, there was an effect of time (Table 3) such that PA increased by ∼10% pre to post‐cast (Figure 5a). For the casted LG, there was a region × joint position × time interaction (Table 3). Pairwise comparisons showed a 26% decrease in PA pre to post‐cast only in distal fascicles at full dorsiflexion (*P* < 0.0001, *d* = 3.24) (Figure 5b).

**FIGURE 6:**
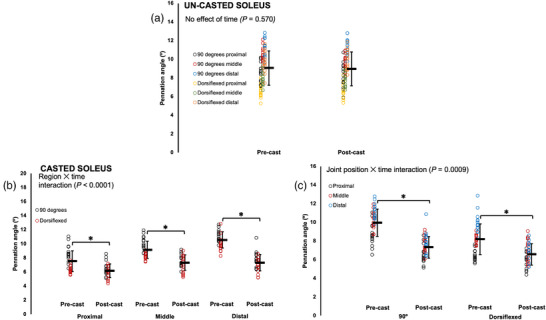
Changes in pennation angle of the un‐casted (a) and casted (b,c) soleus from pre to post‐cast in *n* = 15 rats. Data are presented as means ± standard deviation. *Significant difference between indicated means (*P* < 0.05).

For the un‐casted soleus, like with FL, time did not affect PA, with no changes pre to post‐cast (Table 3; Figure 6a). For the casted soleus, there were interactions of region × time and joint position × time (Table 3). Pairwise comparisons showed that at all regions of muscle, and both joint angles, PA of the casted soleus decreased (9–31%) pre to post‐cast (*P* < 0.0001–0.0005, *d* = 1.08–2.78) (Figure 6b,c).

**FIGURE 7:**
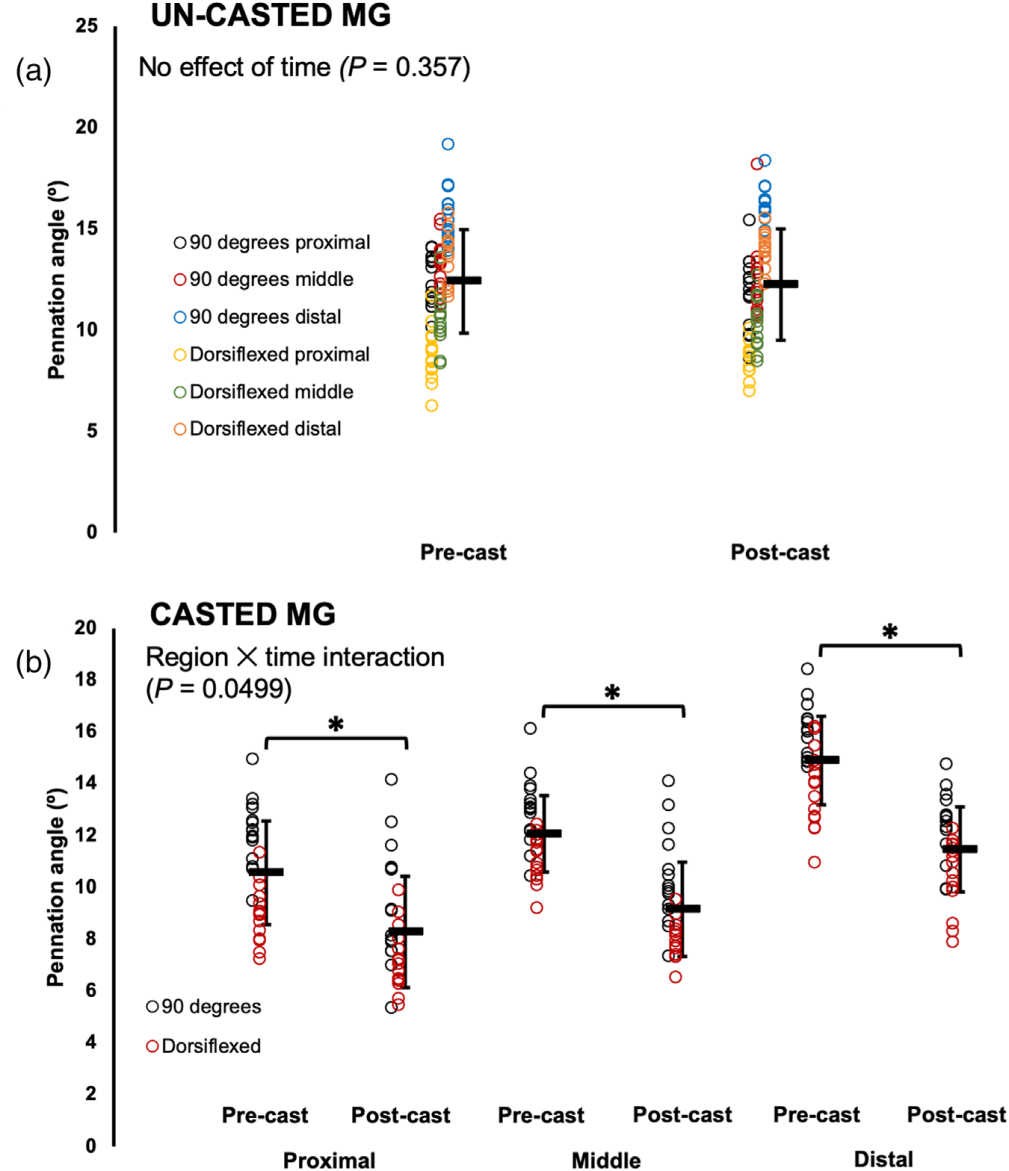
Changes in pennation angle of the un‐casted (a) and casted (b) medial gastrocnemius (MG) from pre to post‐cast in *n* = 15 rats. Data are presented as mean ± standard deviation. *Significant difference between indicated means (*P* < 0.05).

For the un‐casted MG, time did not affect PA, with no changes pre to post‐cast (Table 3; Figure 7a). For the casted MG, there was a region × time interaction (Table 3), and pairwise comparisons showed that at all three regions of the muscle, PA decreased by ∼20% pre to post‐cast (*P* < 0.0001–0.0003, *d* = 1.10–2.05) (Figure 7b).

In summary, PA decreased in the casted soleus and MG from pre to post‐cast and did not change in the un‐casted soleus and MG. For the casted LG, PA decreased from pre to post‐cast, but increased in the un‐casted LG.

### 3.3 Muscle wet weight in the casted versus un‐casted leg

**FIGURE 8:**
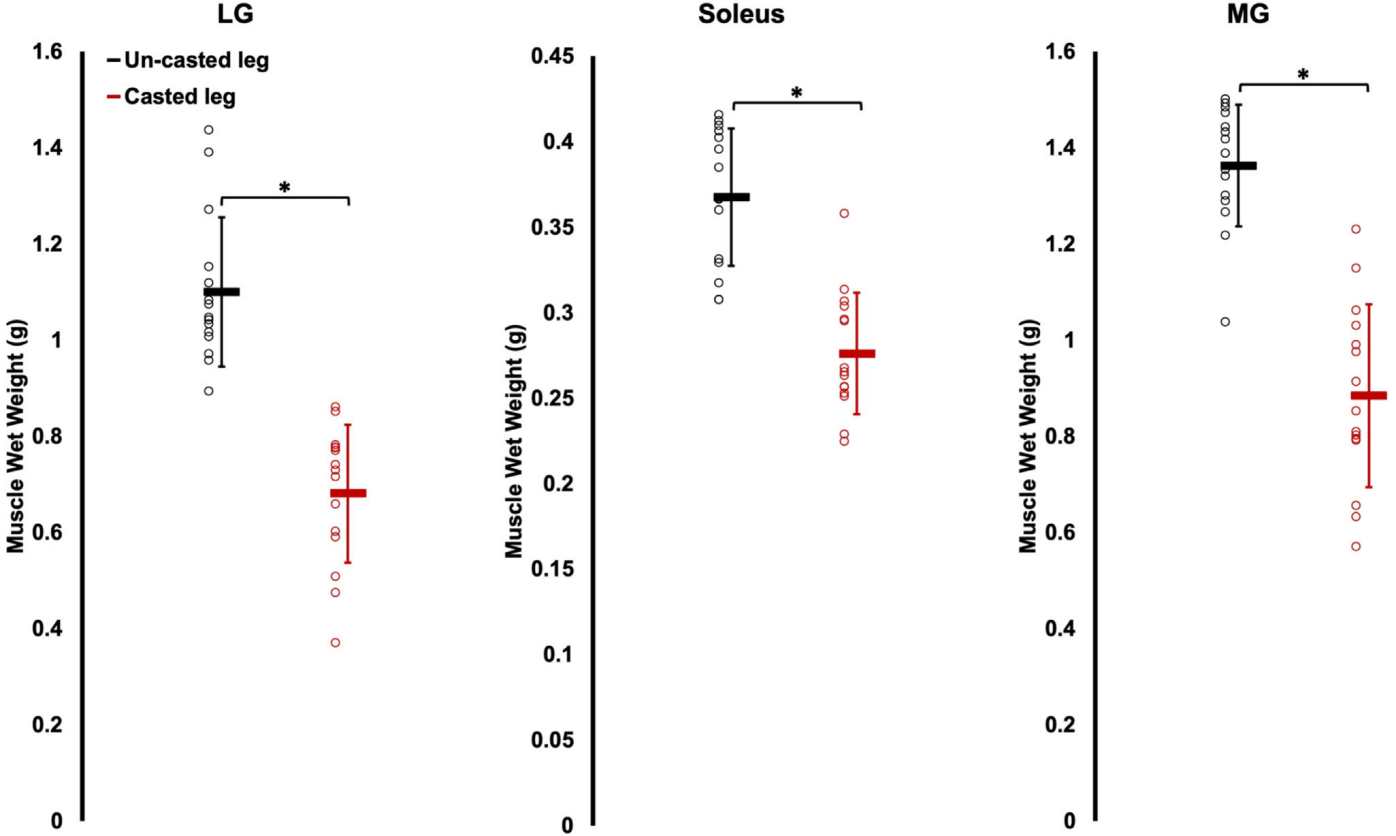
Comparison of muscle wet weight between the casted and un‐casted leg for the lateral gastrocnemius (LG), soleus, and medial gastrocnemius (MG) from *n* = 15 rats. Data are presented as means ± standard deviation. *Significant difference between indicated means (*P* < 0.05).

The LG, soleus and MG of the casted leg weighed 62%, 33% and 54% less, respectively, than the muscles of the un‐casted leg (*P* < 0.0001, *d* = 2.42–2.96) (Figure 8).

### 3.4 Serial sarcomere number, sarcomere length and fascicle length of the dissected fascicles in the casted versus non‐casted leg

Two‐way ANOVA results for SSN, SL and FL of the dissected fascicles are shown in Table 4. There were no region × leg interactions for any muscles.

**TABLE 4:** Two‐way ANOVA results for serial sarcomere number, sarcomere length and fascicle length of dissected fascicles (*n* = 15 rats).

		Effect of region		Effect of leg		Region × leg interaction	
		*F*	*P*	*F*	*P*	*F*	*P*
LG	SSN	108.91	<0.0001*	4.57	0.0507	0.67	0.502
	SL	61.46	<0.0001*	8.37	0.0118*	2.77	0.0959
	FL	55.00	<0.0001*	11.57	0.0043*	0.17	0.793
Soleus	SSN	1.39	0.267	33.02	<0.0001*	0.68	0.475
	SL	1.26	0.291	0.14	0.713	0.64	0.514
	FL	3.06	0.0639	22.94	0.0003*	0.10	0.871
MG	SSN	21.30	<0.0001*	0.99	0.336	1.01	0.374
	SL	18.36	<0.0001*	6.27	0.0253*	1.02	0.373
	FL	7.77	0.0111*	5.03	0.0416*	0.62	0.541

Abbreviations: LG, lateral gastrocnemius; MG, medial gastrocnemius. *Significant effect (*P* < 0.05).

**FIGURE 9:**
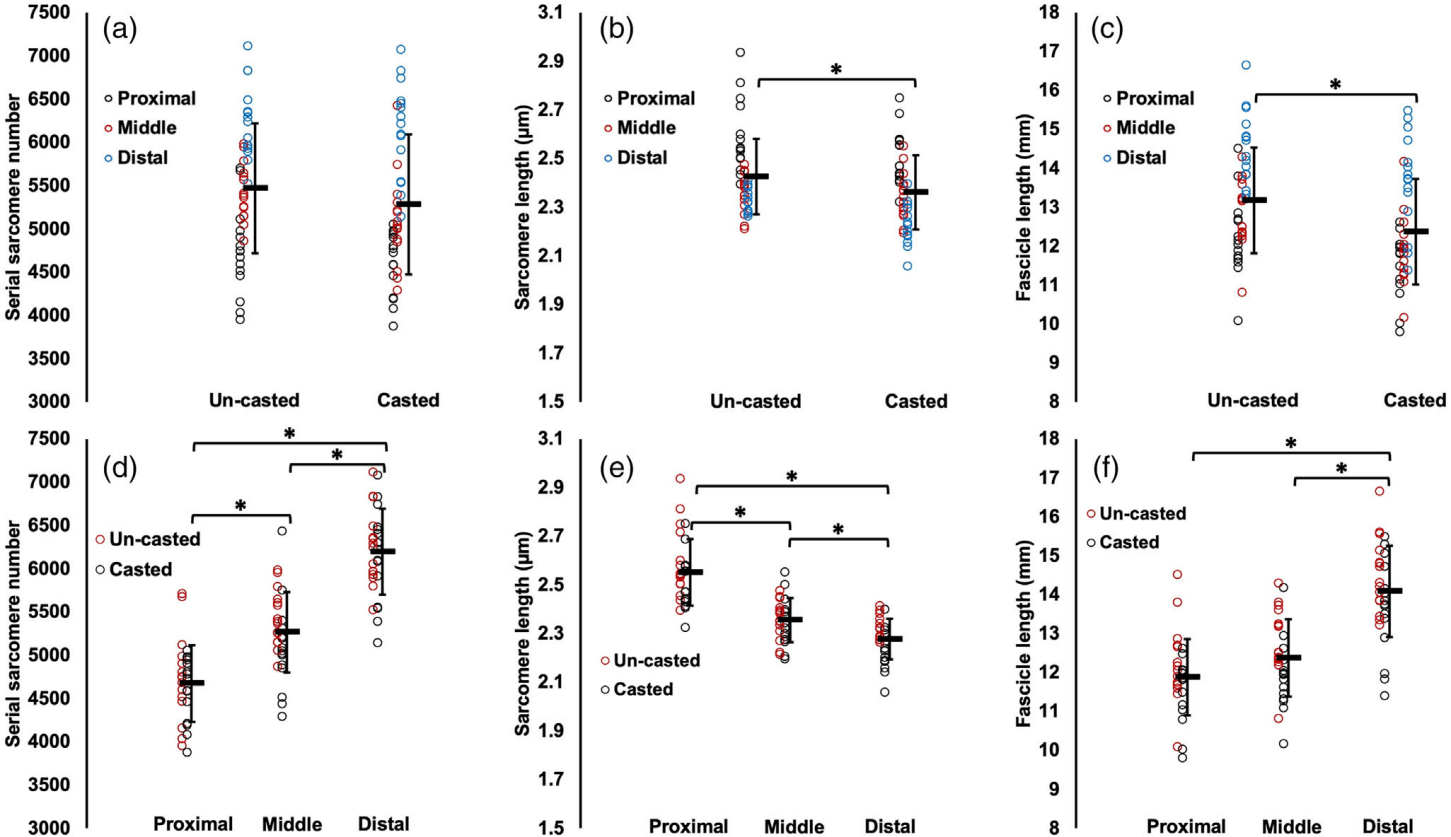
Effects of time (a–c) and effects of region (d–f) on serial sarcomere number (a,d), average sarcomere length (b,e) and fascicle length (c,f) of the lateral gastrocnemius from dissected fascicles in *n* = 15 rats. Data are presented as means ± standard deviation. *Significant difference between indicated means (*P* < 0.05).

For the LG, there were effects of leg (Table 4) on SL and FL of dissected fascicles such that they were 3% and 6% shorter, respectively, in the casted LG (Figure 9b,c). SSN did not differ between the casted and un‐casted LG (Figure 9a). There were effects of region on SSN, SL and FL. SSN increased from proximal to middle to distal (*P* < 0.0001, *d* = 1.30–3.24) (Figure 9d), and FL followed a similar trend (*P* < 0.0001, *d* = 1.58–2.04) but with no difference between proximal and middle FL (*P* = 0.0521) (Figure 9f). Conversely, SL decreased from proximal to middle to distal (*P* < 0.0001–0.0016, *d* = 0.90–2.41) (Figure 9e).

**FIGURE 10:**
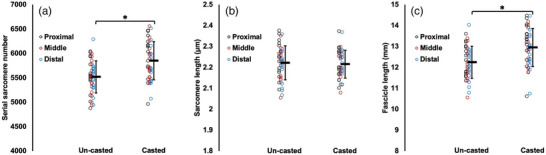
Effects of time on serial sarcomere number (a), average sarcomere length (b) and fascicle length (c) of the soleus from dissected fascicles in *n* = 15 rats. Data are presented as mean ± standard deviation. *Significant difference between indicated means (*P* < 0.05).

For the soleus, there were no effects of region on SSN, SL or dissected FL, indicating no regional differences (Table 4). There was an effect of leg on soleus SSN (Table 4) such that SSN was 6% greater in the casted leg (Figure 10a). There was a similar effect of leg on FL (Table 4), with a 6% increase (Figure 10c). Soleus SL did not differ between the casted and un‐casted leg (Table 4; Figure 10b).

**FIGURE 11:**
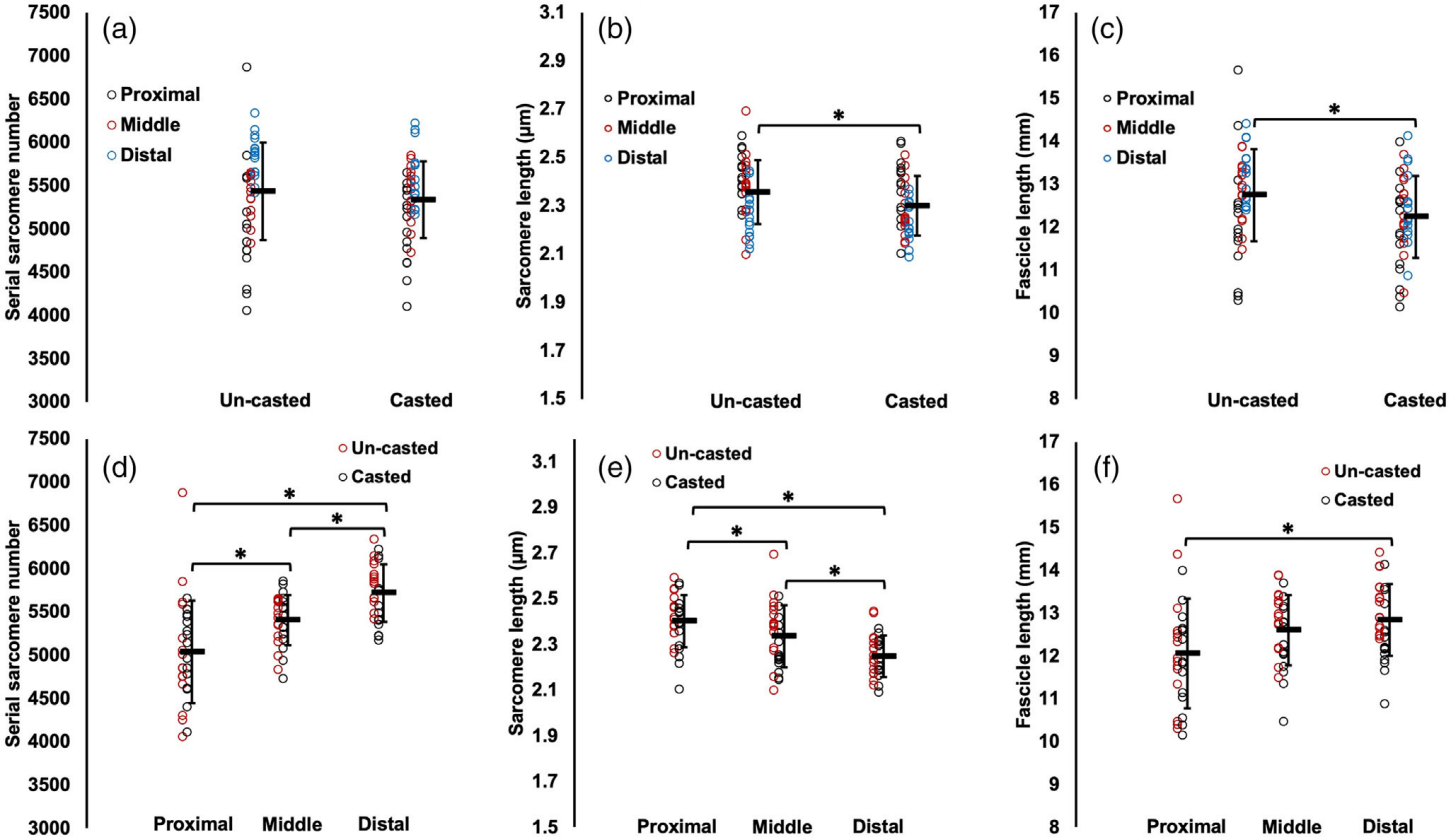
Effects of time (a–c) and effects of region (d–f) on serial sarcomere number (a,d), average sarcomere length (b,e), and fascicle length (c,f) of the medial gastrocnemius from dissected fascicles in *n* = 15 rats. Data are presented as means ± standard deviation. *Significant difference between indicated means (*P* < 0.05).

For the MG, there were effects of leg on SL and dissected FL (Table 4) such that they were 2% and 4% shorter, respectively, in the casted MG (Figure 11b,c). SSN did not differ between the casted and un‐casted MG (Figure 11a). There were effects of region on SSN, SL and FL (Table 4). SSN followed the same pattern as in the LG, increasing from proximal to middle to distal (*P* < 0.0001–0.0127, *d* = 0.79–1.42) (Figure 11d). FL only differed between proximal and distal fascicles, with distal fascicles being longer (*P* = 0.0044, *d* = 0.43) (Figure 11f). Like with the LG, SL decreased from proximal to middle to distal (*P* < 0.0001–0.0481, *d* = 0.52–1.49) (Figure 11e).

In summary, we observed greater SSN in the casted compared to un‐casted soleus, but no differences in SSN between legs were observed for the LG or MG.

### 3.5 Relationships between adaptations in fascicle length measured via ultrasound and adaptations in serial sarcomere number and fascicle length measured from dissected fascicles

**FIGURE 12:**
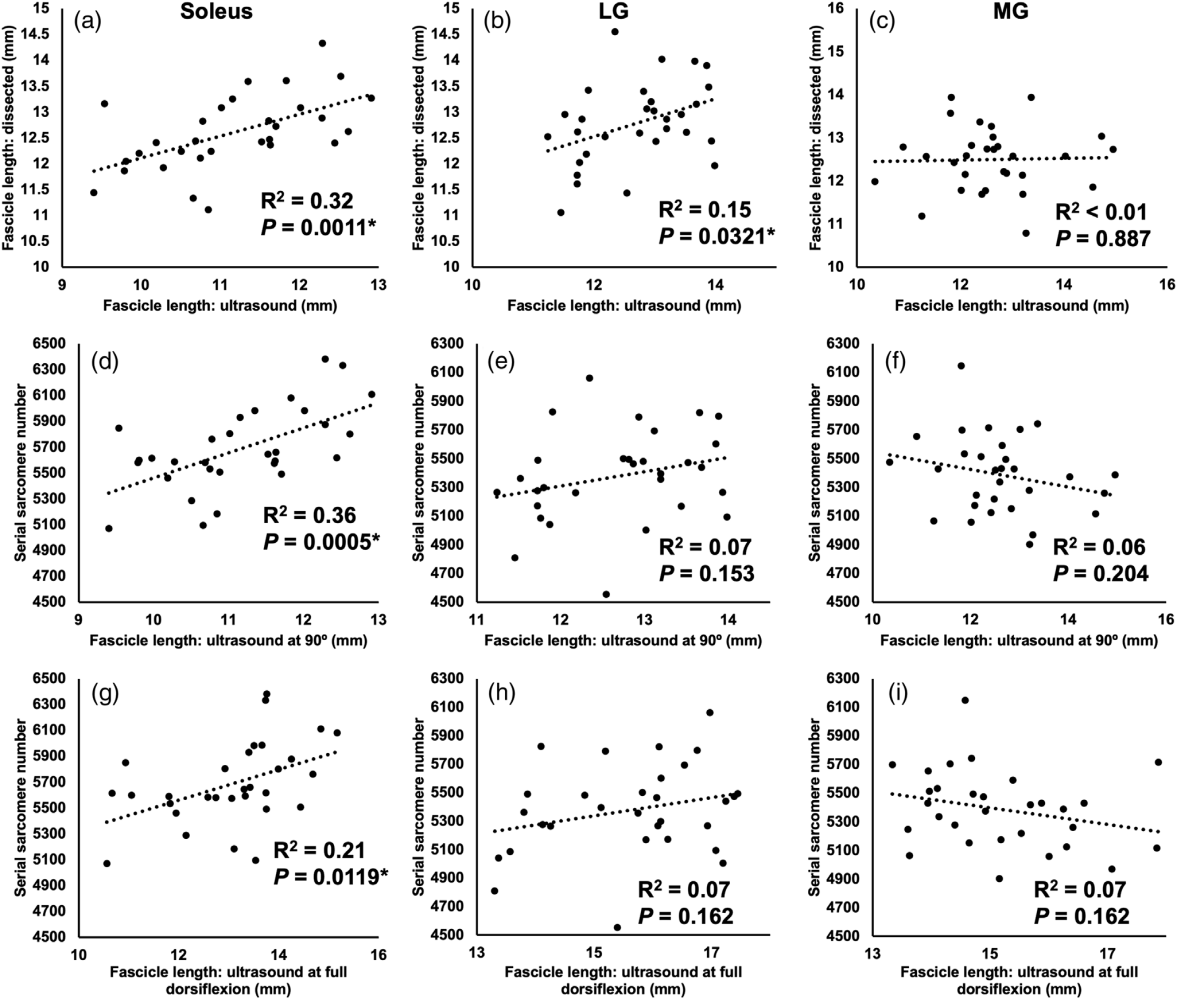
Relationships between ultrasound‐derived fascicle length at 90° and fascicle length of dissected fascicles (a–c), ultrasound‐derived fascicle length at 90° and SSN (d–f), and ultrasound‐derived FL at full dorsiflexion and SSN (g–i) for the soleus, lateral gastrocnemius (LG) and medial gastrocnemius (MG). Each graph displays data from a total of *n* = 30 muscles (*n* = 15 right leg, *n* = 15 left leg) from *n* = 15 rats. *Significant relationship (*P* < 0.05).

For the soleus, significant positive relationships were found between ultrasound‐derived FL at 90° and FL of dissected fascicles (Figure 12a) and SSN (Figure 12d), and between ultrasound‐derived FL at full dorsiflexion and SSN (Figure 12g). For the LG, there was only a relationship between ultrasound‐derived FL at 90° and FL of dissected fascicles (Figure 12b), and no relationships among these measures were observed for the MG (Figure 12c, f, i).

There were no relationships between the percentage change in ultrasound‐derived FL from pre to post‐cast and the percentage change in SSN from the un‐casted to casted leg for the LG, soleus or MG (Table 5).

**TABLE 5:** Relationships between percentage change in ultrasound‐derived fascicle length (FL) from pre to post‐cast and percentage change in serial sarcomere number of dissected fascicles from the un‐casted to casted leg (*n* = 15 rats).

		Ultrasound‐derived FL at 90°		Ultrasound‐derived FL at full dorsiflexion	
		*R* ^2^	*P*	*R* ^2^	*P*
Serial sarcomere number	LG	0.04	0.462	0.02	0.659
	Soleus	0.13	0.180	0.06	0.381
	MG	0.01	0.666	0.03	0.547

Abbreviations: LG, lateral gastrocnemius; MG, medial gastrocnemius.

**FIGURE 13:**
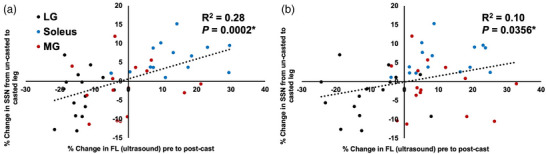
For all muscles combined, relationships between the percentage change in ultrasound‐derived fascicle length (FL) from pre to post‐cast as measured with the ankle at 90° (a) and full dorsiflexion (b) and the percentage change in serial sarcomere number (SSN) from the un‐casted to casted leg determined from dissected fascicles. Each graph displays data from a total of *n* = 45 muscles (*n* = 15 of each of the lateral gastrocnemius (LG), soleus and medial gastrocnemius (MG)) from *n* = 15 rats. *Significant relationship (*P* < 0.05).

When regression analyses were performed across all muscles together, the percentage change in ultrasound‐derived FL measured with the ankle at 90° explained 28% of the variation in the percentage change in SSN from dissected fascicles (Figure 13a). This relationship was lessened when using ultrasound‐derived FL at full dorsiflexion, only explaining 10% of the variation in SSN adaptations (Figure 13b).

## 4 DISCUSSION

Immobilizing the rat ankle in full dorsiflexion for 2 weeks to position the plantar flexor muscles in a lengthened position, the present study investigated whether ultrasound‐derived FL measurements can accurately depict SSN adaptations. Ultrasound detected an 11% increase in soleus FL, a 12% decrease in LG FL, and (depending on the joint angle and region of muscle) an 8% increase in MG FL. These adaptations were partly reflected by the SSN measurements obtained from dissected fascicles, with a 6% greater soleus SSN in the casted leg than the un‐casted leg, but no differences in SSN for the gastrocnemii. Our results indicate that ultrasonographic measurements of FL can overestimate SSN adaptations.

Our values for muscle wet weight, SSN, SL, FL and ultrasound‐derived PA are within previously reported ranges for the rat soleus (Booth, 1977; Chen et al., 2020; Hinks, Jacob et al., 2022; Mele et al., 2016; Peixinho et al., 2011; Soares et al., 2007) and gastrocnemii (Booth, 1977; Mele et al., 2016; Ochi et al., 2007; Peixinho et al., 2011; Power et al., 2021; Woittiez et al., 1986).

### 4.1 Stretch‐induced adaptation in serial sarcomere number

The soleus of the casted leg had a 6% greater SSN than the un‐casted leg, which is consistent with findings from previous studies that immobilized the soleus in a stretched position in rats, mice and cats (Kinney et al., 2017; Shah et al., 2001; Soares et al., 2007; Spector et al., 1982; Tabary et al., 1972; Williams & Goldspink, 1978). This serial sarcomere addition is believed to occur to restore optimal actin–myosin overlap and reduce passive tension in the stretched position (Davis et al., 2020; Hinks, Franchi et al., 2022; Williams & Goldspink, 1978). The increase in rat soleus SSN we observed after 2 weeks of immobilization was notably lower (+6%, from 5518 to 5850 sarcomeres) than the increase reported by Soares et al. (2007) after immobilizing the soleus in a stretched position for just 4 days (+29%, 6338 to 8174 sarcomeres). This discrepancy may be attributed to the more extreme ankle angle they used for immobilization, described as ‘total dorsiflexion’, while we immobilized the ankle at 40°. Additionally, we did not observe a difference in SSN between the casted and un‐casted legs for the gastrocnemii. The gastrocnemii are biarticular muscles, crossing the ankle and the knee. Spector et al. (1982) immobilized the knee in full extension and the ankle at 45°, and observed a 20% increase in MG SSN. While we immobilized the ankle at a similar angle (40°), we did not immobilize the knee, allowing movement of the gastrocnemii at that joint, which likely tempered the stretch stimulus imposed by dorsiflexion for those muscles. Woittiez et al. (1985) showed that, even when at an ankle angle as small as 26°, the knee angle would need to be greater than 90° for the gastrocnemii to be stretched onto the descending limb of their force–length relationships. With that in mind, the lack of a SSN increase and the considerable muscle atrophy (discussed below) we observed in the gastrocnemii are not surprising.

### 4.2 Immobilization‐induced atrophy

Our casting intervention induced atrophy in the gastrocnemii and soleus, as evidenced by lower muscle wet weights in the casted leg. Measurements of PA provided by ultrasound align with these findings, showing decreased PA from pre to post‐cast in all three muscles, which may reflect the loss of sarcomeres in parallel (Narici et al., 2016; Wisdom et al., 2015). The reduced muscle weight was more pronounced in the gastrocnemii (−54% to −62%) than the soleus (−33%). Considering the soleus had a 6% greater SSN in the casted than the un‐casted leg, an increase in SSN due to stretch may have lessened the overall loss of muscle tissue, limiting the loss to only sarcomeres in parallel. A similar result was observed by Spector et al. (1982), with a smaller reduction in soleus wet weight when immobilizing in a stretched position (−14%) than a shortened position (−48%), and the former increasing SSN while the latter decreased SSN. The gastrocnemii in the present study did not appear to experience an increase in SSN, and therefore the loss of parallel sarcomeres may not have been made up for by stretch‐induced serial sarcomere addition, resulting in a greater loss of muscle wet weight.

### 4.3 Can the un‐casted leg be used as a valid control?

In the un‐casted soleus, no differences in FL or PA were detected by ultrasound from pre to post‐cast, validating the use of the un‐casted soleus as a SSN control in the present study and previous studies (Gomes et al., 2004; Heslinga & Huijing, 1993; Kinney et al., 2017; Shah et al., 2001; Williams & Goldspink, 1978). In the un‐casted gastrocnemii, however, ultrasound measurements suggest some adaptations may have occurred, possibly due to the un‐casted leg compensating for the added load (the cast) on the opposite leg during ambulation. In the un‐casted LG, ultrasound showed a 6% decrease in FL at 90°, accompanied by a 10% increase in PA. Increased PA and sometimes a decrease in FL are often observed following training emphasizing concentric contractions, and may reflect a reorganization of the muscle architecture to add sarcomeres in parallel for greater force production (Butterfield et al., 2005; Franchi et al., 2014).

### 4.4 Ultrasound‐derived FL does not perfectly reflect adaptations in serial sarcomere number

Measurements of FL via ultrasound are often used to infer increases or decreases in SSN (Blazevich et al., 2007; Franchi et al., 2014; Hinks et al., 2021; Narici et al., 2003). Inferring SSN adaptations from ultrasound‐derived FL may be problematic, however, because apparent changes in FL may simply be due to changes in SL at the joint angle at which ultrasound measurements are obtained. For example, Pincheira et al. (2021) observed an increase in biceps femoris FL following 3 weeks of eccentric training as measured with the leg in full extension; however, microendoscopy revealed the increase in FL was only due to longer SLs at that joint angle, not training‐induced serial sarcomere addition. In research on animals, SSN adaptations are often determined by calculating SSN from measurements of SL and FL from dissected fascicles, then comparing between experimental and control muscles. The present study investigated the relationship between these two most commonly used methodologies for assessing SSN adaptations.

We observed significant but weak relationships between ultrasound‐derived FL at 90° and FL of dissected fascicles (after being fixed at 90°) for the soleus and LG (Figure 12a,b). Kellis et al. (2009) observed moderate to strong relationships between FL measured via ultrasound and FL measured directly in the hamstrings of human cadavers. Our results may differ from theirs because, after digesting the muscles in nitric acid, it was more difficult to ensure that the same fascicles as in the ultrasound images were being measured from the dissected muscle, even though the same regional constraints (two fascicles from each of the proximal, middle and distal regions) were followed. Additionally, we observed relationships between ultrasound‐derived measurements of FL and actual SSN determined from dissected fascicles for the soleus only, and between the percentage change in FL from pre to post‐cast and the percentage change in SSN from the un‐casted to casted leg with all muscles together. In both cases, the relationships using FL measured at full dorsiflexion were weaker than when using FL measured at 90°. Similar findings were observed recently by Werkhausen et al. (2023), with the relationship between ultrasound‐derived FL and isokinetic force (i.e., a measure associated with SSN; Drazan et al., 2019; Hinks, Franchi et al., 2022) being moderate or non‐existent depending on the joint angle used during ultrasound imaging. Collectively, our regression analyses demonstrate variability both among muscles and between joint angles in the ability for ultrasound‐derived FL to truly represent SSN.

Overall, the soleus provided the best means for comparing ultrasound‐derived FL adaptations and adaptations in SSN, as the un‐casted soleus did not appear to undergo any compensatory adaptations. From the ultrasound measurements, we observed an ∼11% increase in soleus FL from pre to post‐cast, but the true increase in SSN from the un‐casted to the casted leg was only 6%. This serial sarcomere addition appeared to be driven by a 6% increase in FL, as the un‐casted and casted soleus had the same SL (∼2.2 μm) with the ankle fixed at 90°. Interestingly, while ultrasound‐derived FL averaged across muscle regions increased by 11%, the increase in ultrasound‐derived FL of proximal fascicles (+6%) was closer to the observed increase in SSN, demonstrating regional variability in the accuracy of ultrasound‐derived FL measurements. Altogether, an increase in FL measured by ultrasound can indeed correspond to an increase in SSN in the rat soleus, but may overestimate the increase in SSN by as much as 5%.

The magnitude of SSN increase can also vary depending on the duration of immobilization such that sarcomeres continue to be added until reaching a plateau (Aoki et al., 2009; Soares et al., 2007). A greater magnitude of stretch imposed on the muscle also appears to induce greater serial sarcomerogenesis (Herbert & Balnave, 1993), and as discussed earlier, this can especially introduce inter‐muscle differences for bi‐articular muscles such as the gastrocnemii if not all associated joints are immobilized (Spector et al., 1982). Additionally, we only investigated an intervention expected to increase SSN. Interventions expected to decrease SSN such as immobilization in a shortened position (Sarto et al., 2021; Tabary et al., 1972; Williams & Goldspink, 1978) or training emphasizing concentric muscle contractions (Butterfield et al., 2005; Franchi et al., 2014; Lynn et al., 1998) are also widely studied. To better understand the potential for ultrasound‐derived FL to overestimate SSN adaptations, further investigation is needed using different interventions and muscle groups. As well, the best method currently for assuming SSN from in vivo ultrasound measurements in humans is by determining optimal FL (via construction of an active torque–angle relationship), which can allow estimation of SSN using a known optimal SL (Hinks, Franchi et al., 2022, 2023; Reeves et al., 2004). Since we did not assess the relationship between actual SSN and ultrasound‐derived FL measured at a pre‐determined optimal joint angle, this would be an important next step for better understanding how in vivo ultrasound measurements relate to muscle mechanical function.

### 4.5 Limitations that may contribute to a disconnect between ultrasound‐derived FL and actual SSN

For the gastrocnemii, the distal fascicles were sometimes partly out of plane (Figure 1), and thus the trajectory of those fascicles to the deep aponeurosis was used to complete the measurements of FL. This limitation may have contributed to the higher coefficients of variation for the gastrocnemii compared to the soleus (Table 1), and could partly explain the lack of relationships observed between ultrasound‐derived FL and dissected FL and SSN for the gastrocnemii, but not the soleus.

It is also important to note that ultrasound images do not capture the contractile tissue (black/dark) of muscle fascicles per se, but rather the perimysium, the sheath of connective tissue (white/bright) surrounding each fascicle. As such, FL in ultrasound images is measured within the context of structural components including the extracellular matrix and intra‐ and extracellular fluid, while these components are absent in FL measurements on dissected fascicles following digestion in nitric acid. During serial sarcomerogenesis, the connective tissue scaffolding must be constructed before sarcomeres are added within that space (Kjær, 2004), and therefore the time course of adaptations may affect the ability of ultrasound‐derived FL to represent SSN. Previous studies have reported no changes (Williams et al., 1988) or increases in intramuscular connective tissue content (Ahtikoski et al., 2001; Jozsa et al., 1988) following immobilization in a stretched position. Connective tissue in the soleus in particular can increase from ∼2% to ∼20% of muscle volume after 2 weeks of immobilization in a stretched position (Jozsa et al., 1988), the same duration used in the present study. Altogether, variability in connective tissue likely contributes to differences between ultrasound‐derived FL and FL (and thus SSN) measured from dissected fascicles.

Furthermore, an ultrasound image only captures a fascicle path in two dimensions, but the three‐dimensional nature of fascicle curvature is well‐documented (Cameron et al., 2023; Raiteri et al., 2016; Rana et al., 2013). Unless methods such as three‐dimensional ultrasound (Raiteri et al., 2016) or magnetic resonance diffusion tensor imaging (Cameron et al., 2023) are used, the three‐dimensional nature of FL can only be accounted for when fascicles are dissected out of the muscle. In the present study, this two‐dimensional limitation of ultrasound is most evident in how dissected FLs of the soleus were ∼13% longer than ultrasound‐derived FLs. There may be curvature in rat soleus fascicles that is not captured in a lateral ultrasound scan, making fascicles appear shorter. Altogether, these factors may have contributed to the disconnects between ultrasound‐derived FL and actual SSN in the present study, including the ∼5% overestimation of sarcomerogenesis in the soleus, and should be considered going forward in studies employing muscle ultrasound.

Lastly, formalin fixation can result in ∼4% shrinkage (Herring et al., 1984; Julian & Sollins, 1975); however, this would only affect SL and FL, but not SSN since that is constant regardless of muscle position. Any shrinkage would also have occurred in both the casted and un‐casted legs, and therefore we would expect the relative differences between them to remain the same. Moreover, as explained by Page (1974), holding the overall length of the muscle constant while in fixative can help reduce shrinkage. Since we fixed the whole leg in formalin, pinning it in place to a silicone‐bottomed container, we minimized the amount of shrinkage that could occur.

### 4.6 Conclusion

The present study investigated the relationship between the two most commonly used methods of assessing longitudinal growth of skeletal muscle: (1) ultrasound‐derived FL measurements pre and post‐intervention; and (2) comparison of SSN between an experimental and a control muscle. We showed that ultrasound‐derived FL overestimated SSN adaptations by ∼5%, with measurements in a neutral position predicting SSN better than measurements in a stretched position. Future studies should consider these findings when concluding that there is a large magnitude of serial sarcomerogenesis based on ultrasound‐derived FL taken at a set joint angle. Further research across different muscles, interventions and species is needed to develop a precise correction factor to more closely approximate actual SSN adaptations from ultrasound‐derived FL measurements.

## AUTHOR CONTRIBUTIONS

Avery Hinks, Martino V. Franchi and Geoffrey A. Power conceived and designed research; Avery Hinks carried out animal husbandry and casting procedures; Avery Hinks performed experiments; Avery Hinks analysed data; Avery Hinks, Martino V. Franchi and Geoffrey A. Power interpreted results of experiments; Avery Hinks prepared figures; Avery Hinks and Geoffrey A. Power drafted manuscript; Avery Hinks, Martino V. Franchi, and Geoffrey A. Power edited and revised manuscript. All authors have read and approved the final version of this manuscript and agree to be accountable for all aspects of the work in ensuring that questions related to the accuracy or integrity of any part of the work are appropriately investigated and resolved. All persons designated as authors qualify for authorship, and all those who qualify for authorship are listed.

## CONFLICT OF INTEREST

No conflicts of interest, financial or otherwise, are declared by the authors.

## Supporting information


**Supplemental Figure S1**: Example of distal fascicles from the right lateral gastrocnemius used for measurement of dissected fascicle length and calculation of serial sarcomere number, with fascicles positioned in the same plane as a ruler used to set the scale.

## Data Availability

Individual values of all supporting data are available upon request.

## References

[eph13414-bib-0001] Adkins, A. N. , Dewald, J. P. A. , Garmirian, L. P. , Nelson, C. M. , & Murray, W. M. (2021). Serial sarcomere number is substantially decreased within the paretic biceps brachii in individuals with chronic hemiparetic stroke. Proceedings of the National Academy of Sciences, USA, 118(26), e2008597118. 10.1073/pnas.2008597118 PMC8256086 34172565

[eph13414-bib-0002] Ahtikoski, A. M. , Koskinen, S. O. A. , Virtanen, P. , Kovanen, V. , & Takala, T. E. S. (2001). Regulation of synthesis of fibrillar collagens in rat skeletal muscle during immobilization in shortened and lengthened positions. Acta Physiologica Scandinavica, 172(2), 131–140. 11442453 10.1046/j.1365-201X.2001.00848.x

[eph13414-bib-0003] Aoki, M. S. , Soares, A. G. , & Miyabara, E. H. , Baptista, I. L. , & Moriscot, A. S. (2009). Expression of genes related to myostatin signaling during rat skeletal muscle longitudinal growth: Myostatin and Longitudinal Growth. Muscle & Nerve, 40(6), 992–999. 19705480 10.1002/mus.21426

[eph13414-bib-0004] Blazevich, A. J. , Cannavan, D. , Coleman, D. R. , & Horne, S. (2007). Influence of concentric and eccentric resistance training on architectural adaptation in human quadriceps muscles. Journal of Applied Physiology, 103(5), 1565–1575. 17717119 10.1152/japplphysiol.00578.2007

[eph13414-bib-0005] Boakes, J. L. , Foran, J. , Ward, S. R. , & Lieber, R. L. (2007). Muscle adaptation by serial sarcomere addition 1 year after femoral lengthening. Clinical Orthopaedics and Related Research, 456, 250–253. 17065842 10.1097/01.blo.0000246563.58091.af

[eph13414-bib-0006] Booth, F. W. (1977). Time course of muscular atrophy during immobilization of hindlimbs in rats. Journal of Applied Physiology, 43(4), 656–661. 198396 10.1152/jappl.1977.43.4.656

[eph13414-bib-0007] Butterfield, T. A. , Leonard, T. R. , & Herzog, W. (2005). Differential serial sarcomere number adaptations in knee extensor muscles of rats is contraction type dependent. Journal of Applied Physiology, 99(4), 1352. 15947030 10.1152/japplphysiol.00481.2005

[eph13414-bib-0008] Cameron, D. , Reiter, D. A. , Adelnia, F. , Ubaida‐Mohien, C. , Bergeron, C. M. , Choi, S. , Fishbein, K. W. , Spencer, R. G. , & Ferrucci, L. (2023). Age‐related changes in human skeletal muscle microstructure and architecture assessed by diffusion‐tensor magnetic resonance imaging and their association with muscle strength. Aging Celle, 22(7), 13851. 10.1111/acel.13851 PMC10352548 37162031

[eph13414-bib-0009] Chen, J. , Mashouri, P. , Fontyn, S. , Valvano, M. , Elliott‐Mohamed, S. , Noonan, A. M. , Brown, S. H. M. , & Power, G. A. (2020). The influence of training‐induced sarcomerogenesis on the history dependence of force. Journal of Experimental Biology, 223(15), jeb218776. 32561632 10.1242/jeb.218776

[eph13414-bib-0010] Davis, J. F. , Khir, A. W. , Barber, L. , Reeves, N. D. , Khan, T. , DeLuca, M. , & Mohagheghi, A. A. (2020). The mechanisms of adaptation for muscle fascicle length changes with exercise: Implications for spastic muscle. Medical Hypotheses, 144, 110199. 33254508 10.1016/j.mehy.2020.110199

[eph13414-bib-0011] de Boer, M. D. , Seynnes, O. R. , di Prampero, P. E. , Pišot, R. , Mekjavić, I. B. , Biolo, G. , & Narici, M. V. (2008). Effect of 5 weeks horizontal bed rest on human muscle thickness and architecture of weight bearing and non‐weight bearing muscles. European Journal of Applied Physiology, 104(2), 401–407. 18320207 10.1007/s00421-008-0703-0

[eph13414-bib-0012] Drazan, J. F. , Hullfish, T. J. , & Baxter, J. R. (2019). Muscle structure governs joint function: Linking natural variation in medial gastrocnemius structure with isokinetic plantar flexor function. Biology Open, 8(12), 048520. 10.1242/bio.048520 PMC6918776 31784422

[eph13414-bib-0013] Franchi, M. V. , Atherton, P. J. , Reeves, N. D. , Flück, M. , Williams, J. , Mitchell, W. K. , Selby, A. , Beltran Valls, R. M. , & Narici, M. V. (2014). Architectural, functional and molecular responses to concentric and eccentric loading in human skeletal muscle. Acta Physiologica (Oxford, England), 210(3), 642–654. 24387247 10.1111/apha.12225

[eph13414-bib-0014] Franchi, M. V. , Fitze, D. P. , Raiteri, B. J. , Hahn, D. , & Spörri, J. (2019). Ultrasound‐derived Biceps femoris long‐head fascicle length: Extrapolation pitfalls. Medicine and Science in Sports and Exercise, 52(1), 233–243. 10.1249/MSS.0000000000002123 31403609

[eph13414-bib-0015] Gomes, A. R. S. , Coutinho, E. L. , França, C. N. , Polonio, J. , & Salvini, T. F. (2004). Effect of one stretch a week applied to the immobilized soleus muscle on rat muscle fiber morphology. Brazilian Journal of Medical and Biological Research, 37(10), 1473–1480. 15448867 10.1590/s0100-879x2004001000005

[eph13414-bib-0016] Herbert, R. D. , & Balnave, R. J. (1993). The effect of position of immobilisation on resting length, resting stiffness, and weight of the soleus muscle of the rabbit. Journal of Orthopaedic Research, 11(3), 358–366. 8326442 10.1002/jor.1100110307

[eph13414-bib-0017] Herring, S. W. , Grimm, A. F. , & Grimm, B. R. (1984). Regulation of sarcomere number in skeletal muscle: A comparison of hypotheses. Muscle & Nerve, 7(2), 161–173. 6717493 10.1002/mus.880070213

[eph13414-bib-0018] Heslinga, J. W. , & Huijing, P. A. (1993). Muscle length‐force characteristics in relation to muscle architecture: A bilateral study of gastrocnemius medialis muscles of unilaterally immobilized rats. European Journal of Applied Physiology, 66(4), 289–298. 10.1007/BF00237771 8495688

[eph13414-bib-0019] Hinks, A. , Davidson, B. , Akagi, R. , & Power, G. A. (2021). Influence of isometric training at short and long muscle‐tendon unit lengths on the history dependence of force. Scandinavian Journal of Medicine & Science in Sports, 31(2), 325–338. 33038040 10.1111/sms.13842

[eph13414-bib-0020] Hinks, A. , Franchi, M. V. , & Power, G. A. (2022). The influence of longitudinal muscle fascicle growth on mechanical function. Journal of Applied Physiology, 133(1), 87–103. 35608202 10.1152/japplphysiol.00114.2022

[eph13414-bib-0021] Hinks, A. , Hawke, T. J. , Franchi, M. V. , & Power, G. A. (2023). The importance of serial sarcomere addition for muscle function and the impact of aging. Journal of Applied Physiology, 135(2), 375–393. 37410905 10.1152/japplphysiol.00205.2023

[eph13414-bib-0022] Hinks, A. , Jacob, K. , Mashouri, P. , Medak, K. D. , Franchi, M. V. , Wright, D. C. , Brown, S. H. M. , & Power, G. A. (2022). Influence of weighted downhill running training on serial sarcomere number and work loop performance in the rat soleus. Biology Open, 11(7), bio059491. 35876382 10.1242/bio.059491 PMC9346294

[eph13414-bib-0023] Jozsa, L. , Thöring, J. , Järvinen, M. , Kannus, P. , Lehto, M. , & Kvist, M. (1988). Quantitative alterations in intramuscular connective tissue following immobilization: An experimental study in the rat calf muscles. Experimental and Molecular Pathology, 49(2), 267–278. 3169207 10.1016/0014-4800(88)90039-1

[eph13414-bib-0024] Julian, F. J. , & Sollins, M. R. (1975). Sarcomere length‐tension relations in living rat papillary muscle. Circulation Research, 37(3), 299–308. 1157219 10.1161/01.res.37.3.299

[eph13414-bib-0025] Kellis, E. , Galanis, N. , Natsis, K. , & Kapetanos, G. (2009). Validity of architectural properties of the hamstring muscles: Correlation of ultrasound findings with cadaveric dissection. Journal of Biomechanics, 42(15), 2549–2554. 19646698 10.1016/j.jbiomech.2009.07.011

[eph13414-bib-0026] Kinney, M. C. , Dayanidhi, S. , Dykstra, P. B. , McCarthy, J. J. , Peterson, C. A. , & Lieber, R. L. (2017). Reduced skeletal muscle satellite cell number alters muscle morphology after chronic stretch but allows limited serial sarcomere addition: Satellite cells and sarcomere addition. Muscle & Nerve, 55(3), 384–392. 27343167 10.1002/mus.25227 PMC5183525

[eph13414-bib-0027] Kjær, M. (2004). Role of extracellular matrix in adaptation of tendon and skeletal muscle to mechanical loading. Physiological Reviews, 84(2), 649–698. 15044685 10.1152/physrev.00031.2003

[eph13414-bib-0028] Lichtwark, G. A. , Farris, D. J. , Chen, X. , Hodges, P. W. , & Delp, S. L. (2018). Microendoscopy reveals positive correlation in multiscale length changes and variable sarcomere lengths across different regions of human muscle. Journal of Applied Physiology, 125(6), 1812–1820. 30212307 10.1152/japplphysiol.00480.2018

[eph13414-bib-0029] Lieber, R. L. , & Fridén, J. (2000). Functional and clinical significance of skeletal muscle architecture. Muscle & Nerve, 23(11), 1647–1666. 11054744 10.1002/1097-4598(200011)23:11<1647::aid-mus1>3.0.co;2-m

[eph13414-bib-0030] Lieber, R. L. , Ljung, B. O. , & Fridén, J. (1997). Intraoperative sarcomere length measurements reveal differential design of human wrist extensor muscles. Journal of Experimental Biology, 200(1), 19–25. 9023992 10.1242/jeb.200.1.19

[eph13414-bib-0031] Lieber, R. L. , Yeh, Y. , & Baskin, R. J. (1984). Sarcomere length determination using laser diffraction. Effect of beam and fiber diameter. Biophysical Journal, 45(5), 1007–1016. 6610443 10.1016/S0006-3495(84)84246-0 PMC1434983

[eph13414-bib-0032] Lynn, R. , Talbot, J. A. , & Morgan, D. L. (1998). Differences in rat skeletal muscles after incline and decline running. Journal of Applied Physiology, 85(1), 98–104. 9655761 10.1152/jappl.1998.85.1.98

[eph13414-bib-0033] Mele, A. , Fonzino, A. , Rana, F. , Camerino, G. M. , De Bellis, M. , Conte, E. , Giustino, A. , Conte Camerino, D. , & Desaphy, J.‐F. (2016). In vivo longitudinal study of rodent skeletal muscle atrophy using ultrasonography. Scientific Reports, 6(1), 20061. 26832124 10.1038/srep20061 PMC4735519

[eph13414-bib-0034] Narici, M. , Franchi, M. , & Maganaris, C. (2016). Muscle structural assembly and functional consequences. Journal of Experimental Biology, 219(2), 276–284. 26792340 10.1242/jeb.128017

[eph13414-bib-0035] Narici, M. V. , Maganaris, C. N. , Reeves, N. D. , & Capodaglio, P. (2003). Effect of aging on human muscle architecture. Journal of Applied Physiology, 95(6), 2229–2234. 12844499 10.1152/japplphysiol.00433.2003

[eph13414-bib-0036] Ochi, E. , Nakazato, K. , & Ishii, N. (2007). Effects of eccentric exercise on joint stiffness and muscle connectin (Titin) isoform in the rat hindlimb. Journal of Physiological Sciences, 57(1), 1–6. 10.2170/physiolsci.RP008806 17081353

[eph13414-bib-0037] Page, S. G. (1974). Measurements of Structural Parameters in Cardiac Muscle. In Ciba Foundation Symposium 24 ‐ Physiological Basis of Starling's Law of the Heart , pp. 13–30. John Wiley & Sons, Ltd.

[eph13414-bib-0038] Peixinho, C. C. , Martins, N. S. F. , de Oliveira, L. F. , & Machado, J. C. (2014). Structural adaptations of rat lateral gastrocnemius muscle–tendon complex to a chronic stretching program and their quantification based on ultrasound biomicroscopy and optical microscopic images. Clinical Biomechanics, 29(1), 57–62. 24309012 10.1016/j.clinbiomech.2013.11.002

[eph13414-bib-0039] Peixinho, C. C. , Ribeiro, M. B. , Resende, C. M. C. , Werneck‐de‐Castro, J. P. S. , de Oliveira, L. F. , & Machado, J. C. (2011). Ultrasound biomicroscopy for biomechanical characterization of healthy and injured triceps surae of rats. Journal of Experimental Biology, 214(22), 3880–3886. 22031753 10.1242/jeb.059808

[eph13414-bib-0040] Pincheira, P. A. , Boswell, M. A. , Franchi, M. V. , Delp, S. L. , & Lichtwark, G. A. (2021). Biceps femoris long head sarcomere and fascicle length adaptations after three weeks of eccentric exercise training. Journal of Sport and Health Science, 11(1), 43–49. 34509714 10.1016/j.jshs.2021.09.002 PMC8847943

[eph13414-bib-0041] Power, G. A. , Crooks, S. , Fletcher, J. R. , Macintosh, B. R. , & Herzog, W. (2021). Age‐related reductions in the number of serial sarcomeres contribute to shorter fascicle lengths but not elevated passive tension. Journal of Experimental Biology, 224(10), jeb242172. 34028517 10.1242/jeb.242172

[eph13414-bib-0042] Power, G. A. , Makrakos, D. P. , Rice, C. L. , & Vandervoort, A. A. (2013). Enhanced force production in old age is not a far stretch: An investigation of residual force enhancement and muscle architecture. Physiological Reports, 1(1), e00004. 24303098 10.1002/phy2.4 PMC3831934

[eph13414-bib-0043] Raiteri, B. J. , Cresswell, A. G. , & Lichtwark, G. A. (2016). Three‐dimensional geometrical changes of the human tibialis anterior muscle and its central aponeurosis measured with three‐dimensional ultrasound during isometric contractions. PeerJ, 4, e2260. 27547566 10.7717/peerj.2260 PMC4974924

[eph13414-bib-0044] Rana, M. , Hamarneh, G. , & Wakeling, J. M. (2013). 3D fascicle orientations in triceps surae. Journal of Applied Physiology, 115(1), 116–125. 23640593 10.1152/japplphysiol.01090.2012

[eph13414-bib-0045] Reeves, N. D. , Narici, M. V. , & Maganaris, C. N. (2004). In vivo human muscle structure and function: Adaptations to resistance training in old age: Muscle adaptations to training in old age. Experimental Physiology, 89(6), 675–689. 15328305 10.1113/expphysiol.2004.027797

[eph13414-bib-0046] Sarto, F. , Monti, E. , Šimunič, B. , Pišot, R. , Narici, M. V. , & Franchi, M. V. (2021). Changes in Biceps Femoris Long Head Fascicle Length after 10‐d Bed Rest Assessed with Different Ultrasound Methods. Medicine and Science in Sports and Exercise, 53(7), 1529–1536. 34127637 10.1249/MSS.0000000000002614 PMC10115490

[eph13414-bib-0047] Shah, S. B. , Peters, D. , Jordan, K. A. , Milner, D. J. , Fridén, J. , Capetanaki, Y. , & Lieber, R. L. (2001). Sarcomere number regulation maintained after immobilization in desmin‐null mouse skeletal muscle. Journal of Experimental Biology, 204(10), 1703–1710. 11316490 10.1242/jeb.204.10.1703

[eph13414-bib-0048] Soares, A. G. , Aoki, M. S. , Miyabara, E. H. , DeLuca, C. V. , & Ono, H. Y. , Gomes, M. D. , & Moriscot, A. S. (2007). Ubiquitin‐ligase and deubiquitinating gene expression in stretched rat skeletal muscle. Muscle & Nerve, 36, 685–693. 17657803 10.1002/mus.20866

[eph13414-bib-0049] Spector, S. A. , Simard, C. P. , Fournier, M. , Sternlicht, E. , & Edgerton’, V. R. (1982). Architectural Alterations of Rat Hind‐Limb Skeletal Muscles Immobilized at Different Lengths. Experimental Neurology, 76(1), 94–110. 7084367 10.1016/0014-4886(82)90104-2

[eph13414-bib-0050] Tabary, J. C. , Tabary, C. , Tardieu, C. , Tardieu, G. , & Goldspink, G. (1972). Physiological and structural changes in the cat's soleus muscle due to immobilization at different lengths by plaster casts. The Journal of Physiology, 224(1), 231–244. 5039983 10.1113/jphysiol.1972.sp009891 PMC1331536

[eph13414-bib-0051] Werkhausen, A. , Gløersen, Ø. , Nordez, A. , Paulsen, G. , Bojsen‐Møller, J. , & Seynnes, O. R. (2023). Linking muscle architecture and function in vivo: Conceptual or methodological limitations? PeerJ, 11, e15194. 37077309 10.7717/peerj.15194 PMC10108853

[eph13414-bib-0052] Williams, P. E. , Catanese, T. , Lucey, E. G. , & Goldspink, G. (1988). The importance of stretch and contractile activity in the prevention of connective tissue accumulation in muscle. Journal of Anatomy, 158, 109–114. 3225214 PMC1261981

[eph13414-bib-0053] Williams, P. E. , & Goldspink, G. (1978). Changes in sarcomere length and physiological properties in immobilized muscle. Journal of Anatomy, 127, 459–468. 744744 PMC1235732

[eph13414-bib-0054] Wisdom, K. M. , Delp, S. L. , & Kuhl, E. (2015). Use it or lose it: Multiscale skeletal muscle adaptation to mechanical stimuli. Biomechanics and Modeling in Mechanobiology, 14(2), 195–215. 25199941 10.1007/s10237-014-0607-3 PMC4352121

[eph13414-bib-0055] Woittiez, R. D. , Baan, G. C. , Huijing, P. A. , & Rozendal, R. H. (1985). Functional characteristics of the calf muscles of the rat. Journal of Morphology, 184(3), 375–387. 4057263 10.1002/jmor.1051840311

[eph13414-bib-0056] Woittiez, R. D. , Heerkens, Y. F. , Huijing, P. A. , Rijnsburger, W. H. , & Rozendal, R. H. (1986). Functional morphology of the M. Gastrocnemius medialis of the rat during growth. Journal of Morphology, 187(2), 247–258. 3959087 10.1002/jmor.1051870210

