# Supplementary material for: Ultrasonographic measurements of fascicle length overestimate adaptations in serial sarcomere number
Source: Exp Physiol. 2023 Aug 23;108(10):1308–24. doi: 10.1113/EP091334 (PMC10988429; doi:10.1113/EP091334)
Supplement: Supplementary file 1 — Supplemental Figure S1: Example of distal fascicles from the right lateral gastrocnemius used for measurement of dissected fascicle length and calculation of serial sarcomere number, with fascicles positioned in the same plane as a ruler used to set the scale. [file EPH-108-1308-s001.pdf]

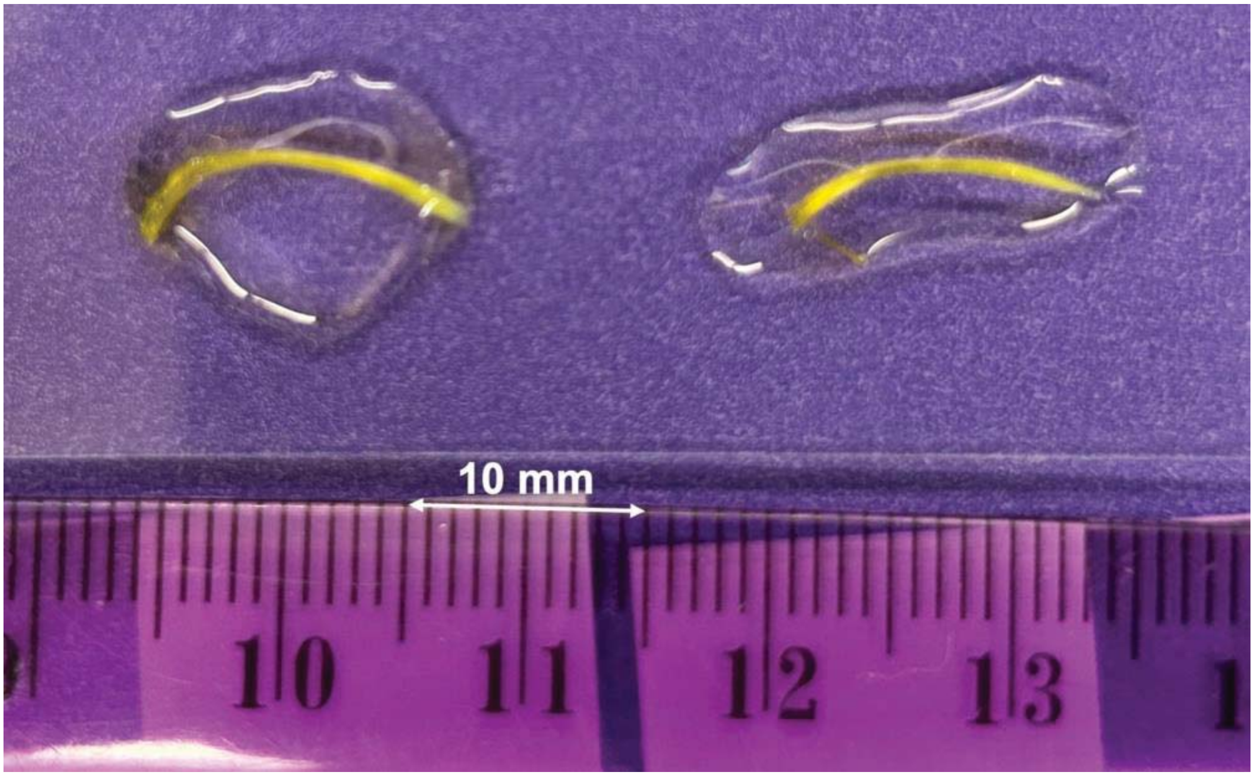

Supplemental Figure S1: Example of distal fascicles from the right lateral gastrocnemius used for measurement of dissected fascicle length and calculation of serial sarcomere number, with fascicles positioned in the same plane as a ruler used to set the scale.
